# Supplementary material for: Supramolecular engineering of charge transfer in wide bandgap organic semiconductors with enhanced visible-to-NIR photoresponse
Source: Nat Commun. 2021 Jun 16;12:3667. doi: 10.1038/s41467-021-23914-2 (PMC8209149; doi:10.1038/s41467-021-23914-2)
Supplement: Supplementary file 1 — Supplementary Information [file 41467_2021_23914_MOESM1_ESM.pdf]

Supplementary Information

Supramolecular engineering of charge transfer in wide bandgap organic semiconductors with enhanced visible-to-NIR photoresponse

Yifan Yao[1], Qi Ou[2], Kuidong Wang[1], Haijun Peng[1], Feier Fang[3], Yumeng Shi[3], Ye Wang[1], Daniel Iglesias Asperilla[1], Zhigang Shuai[2] and Paolo Samorì[1*]

[1] ISIS & icFRC, University of Strasbourg & CNRS, 8 allée Gaspard Monge, 67000 Strasbourg, France.

[2] Department of Chemistry, Tsinghua University, Beijing 100084, China.

[3] Institute of Microscale Optoelectronics, Shenzhen University, Shenzhen 518060, China.

Table of Contents

1. Preparation and characterization of the supramolecular nanowires…..............................…...S2
2. UV-NIR absorption spectrum and AFM images of PTCDI-C8 film prepared by thermal evaporation……………………………………………………………………….…..S3
3. Planar phototransistors of PTCDI-C8 nanowires network deposited by vacuum-assisted stamp method……………………………………………………………………........……S4
4. Planar phototransistors of PTCDI-C8 film prepared by thermal evaporation........................S5
5. Theoretical calculation of charge transfer within PTCDI-C8 nanowire...............................S5
6. SEM and AFM characterizations of nanomesh scaffold on Al modified substrate……........S7
7. Vertical devices prepared by supramolecular nanowires...........................……….......…S7
8. Vertical phototransistors of supramolecular network based on nanomesh scaffold……......S11
9. Vertical phototransistors of supramolecular network based on continuous gold film……..S16
10. References……………………………………………………………………..……...S18

1. **Preparation and characterization of the supramolecular nanowires**

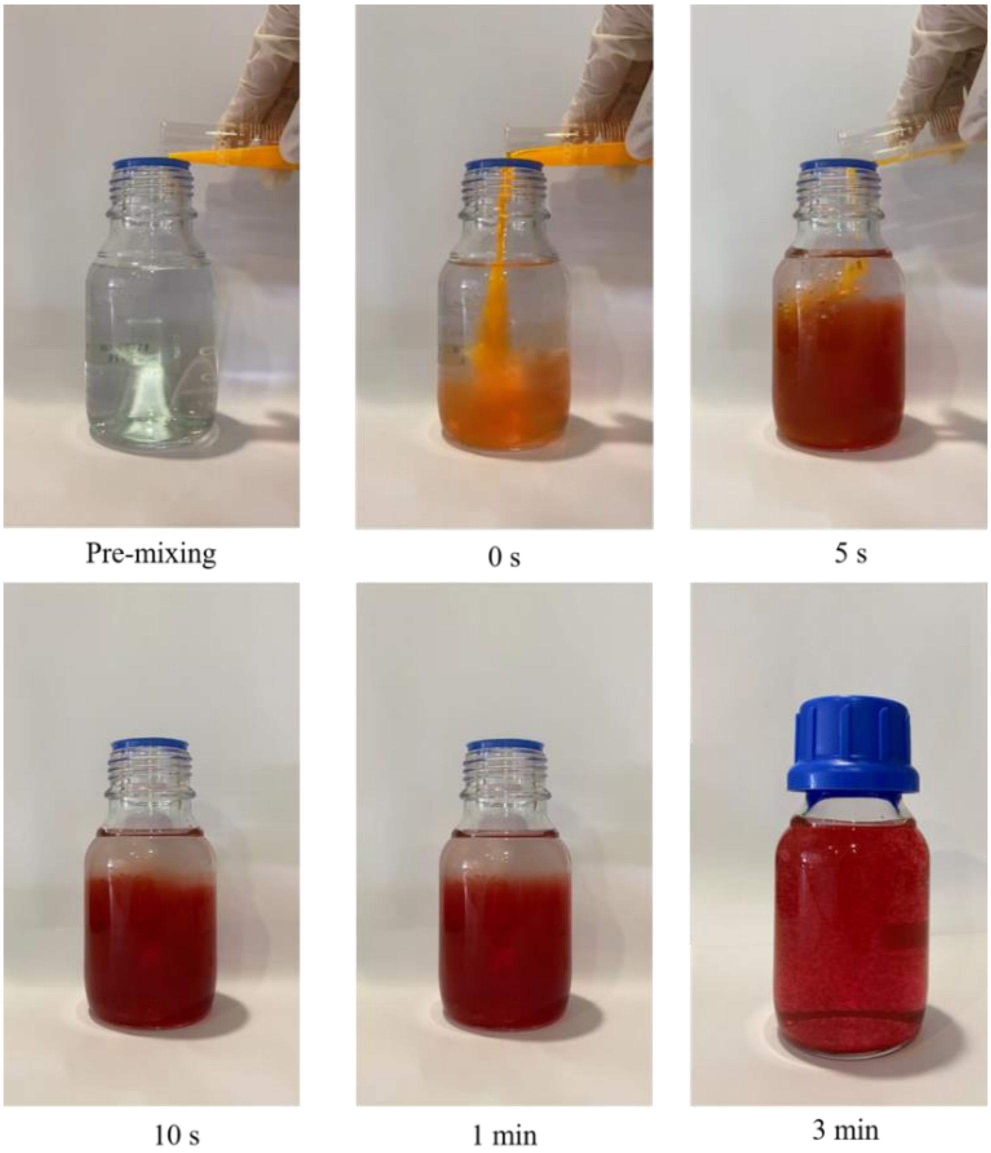

Supplementary Figure 1. A series of optical photographs detailing the growth process of PTCDI-C8 supramolecular nanowires by simply mixing PTCDI-C8/chloroform solution (0.25 mg/ml) with ethanol.

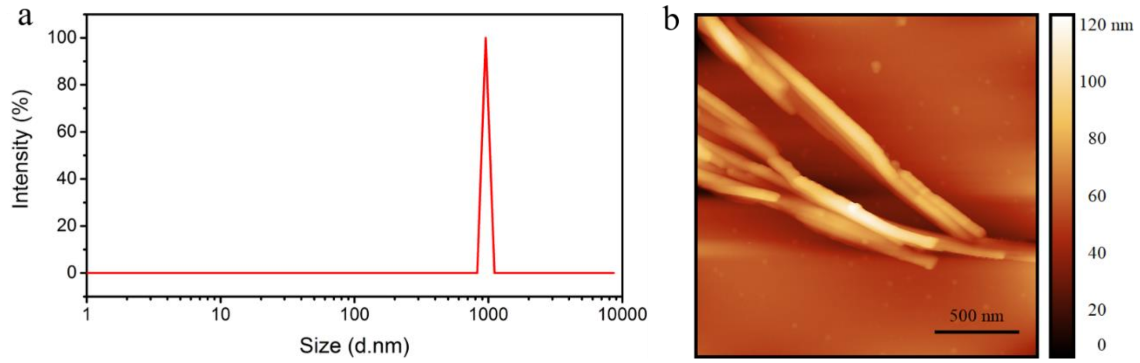

Supplementary Figure 2. a, Dynamic light scattering (DLS) analysis of PTCDI-C8 supramolecular nanowires suspension in ethanol. b, AFM image of PTCDI-C8 supramolecular nanowires after spin-coated on SiO_2/Si substrate.

2. UV-NIR absorption spectrum and AFM images of PTCDI-C8 film prepared by thermal evaporation

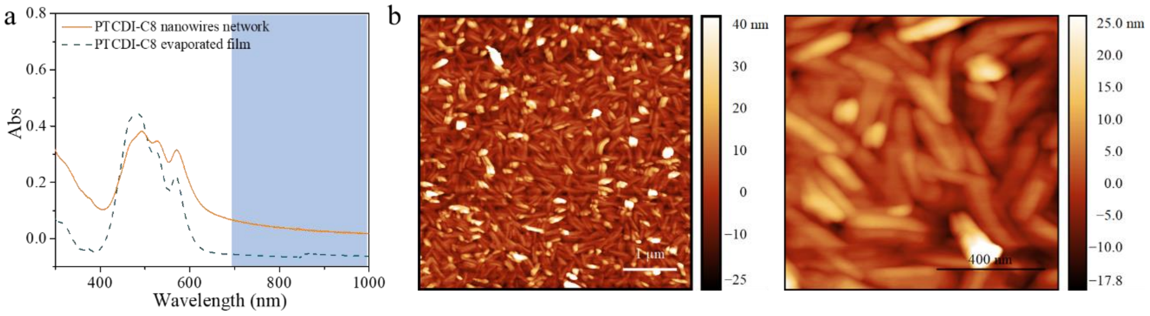

Supplementary Figure 3. a, UV-NIR absorption spectrum of thermal evaporated PTCDI-C8 film and PTCDI-C8 nanowires network on the quartz substrate. b, AFM images of thermal evaporated PTCDI-C8 film with around 200 nm sized rod-like grains.

3. Planar phototransistors of PTCDI-C8 nanowires network deposited by vacuum-assisted stamp method

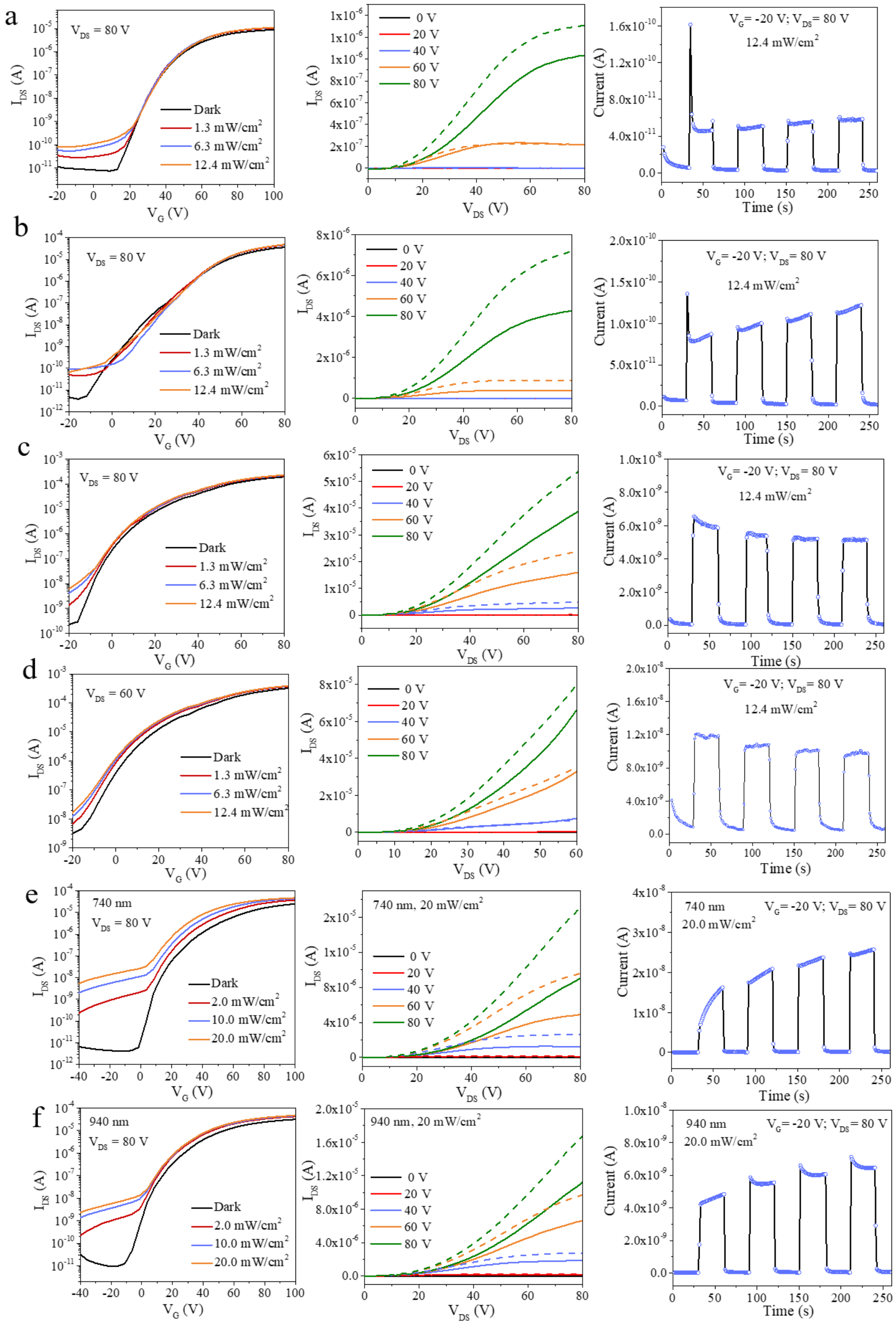

Supplementary Figure 4. Transfer curve, output characteristics (solid line: dark; dashed line: 850 nm, 12.4 mW/cm^2) and optoelectrical switching behavior for the bottom-gate bottom-contact planar phototransistors with PTCDI-C8 nanowires network as the active layer, under 850 nm light illumination. Channel length: a, 20 µm; b, 10 µm; c, 5 µm; d, 2.5 µm. e, f, PTCDI-C8 nanowires network as the active layer, under 740 nm and 940 nm light illumination with channel length of 10 µm.

4. Planar phototransistors of PTCDI-C8 film prepared by thermal evaporation

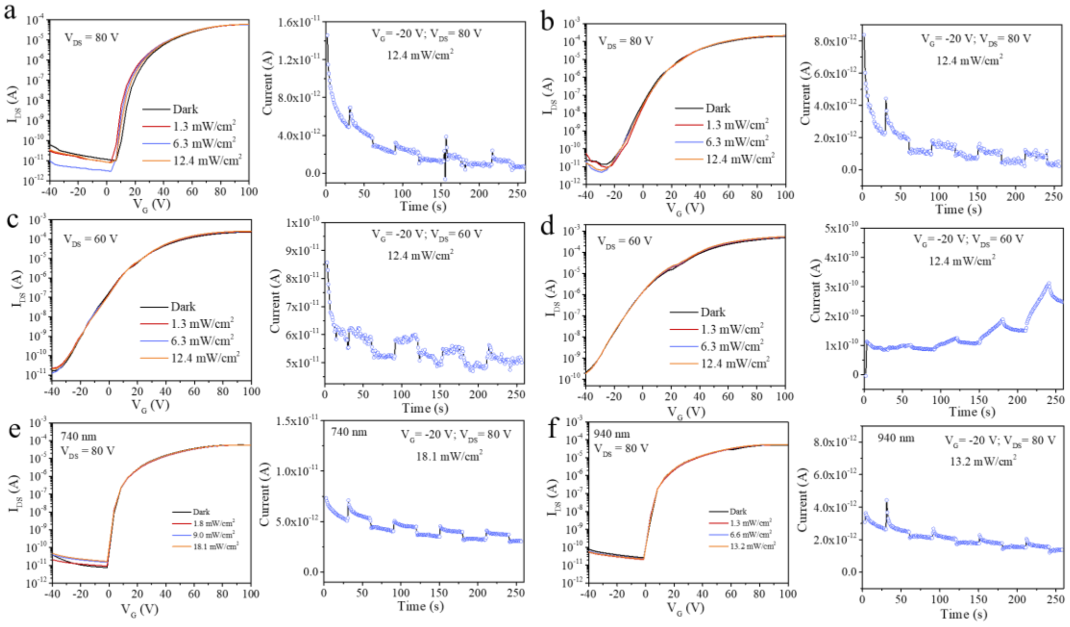

Supplementary Figure 5. Transfer curve and optoelectrical switching behavior for the bottom-gate bottom-contact planar phototransistors with thermal evaporated PTCDI-C8 film as the active layer, under 850 nm light illumination. Channel length: a, 20 µm; b, 10 µm; c, 5 µm; d, 2.5 µm. e, f, Thermally evaporated PTCDI-C8 film as the active layer, under 740 nm and 940 nm light illumination with channel length of 10 µm.

5. Theoretical calculation of charge transfer within PTCDI-C8 nanowire

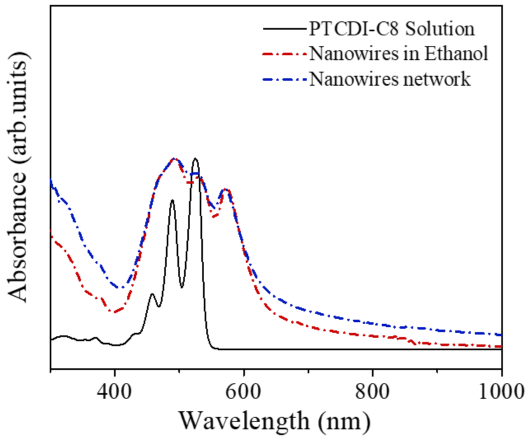

Supplementary Figure 6. UV-NIR absorption spectrum of PTCDI-C8 chloroform solution, PTCDI-C8 nanowire ethanol suspension and PTCDI-C8 nanowires network on the quartz substrate.

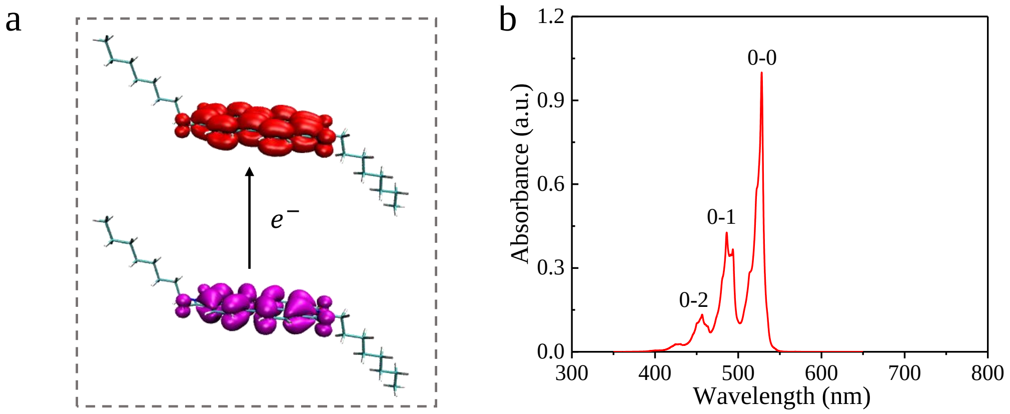

Supplementary Figure 7. a, Attachment-detachment density of the S_1 state of PTCDI-C8 in solution. Red denotes the attachment density and magenta denotes the detachment density. b, Calculated absorption spectrum of PTCDI-C8 in solution.

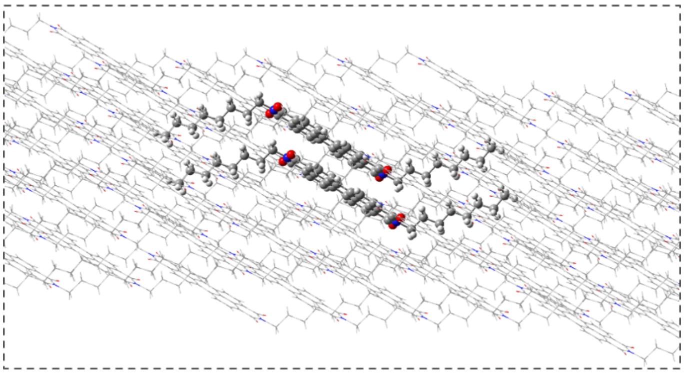

Supplementary Figure 8. Quantum mechanics and molecular mechanics (QM/MM) model for PTCDI-C8 nanowire. A dimer of PTCDI-C8 is chosen inside the crystalline structure and treated as the QM unit, while surrounding molecules are treated as the MM part.

	Absorption energy (eV)	Wavelength (nm)
In solution	2.2769	545
Crystalline structure	2.1119	587

Supplementary Table 1. Theoretical predicted absorption energy and corresponding wavelength of the S_1 state of PTCDI-C8 in solution and in the crystalline structure.

6. SEM and AFM characterizations of nanomesh scaffold on Al modified substrate

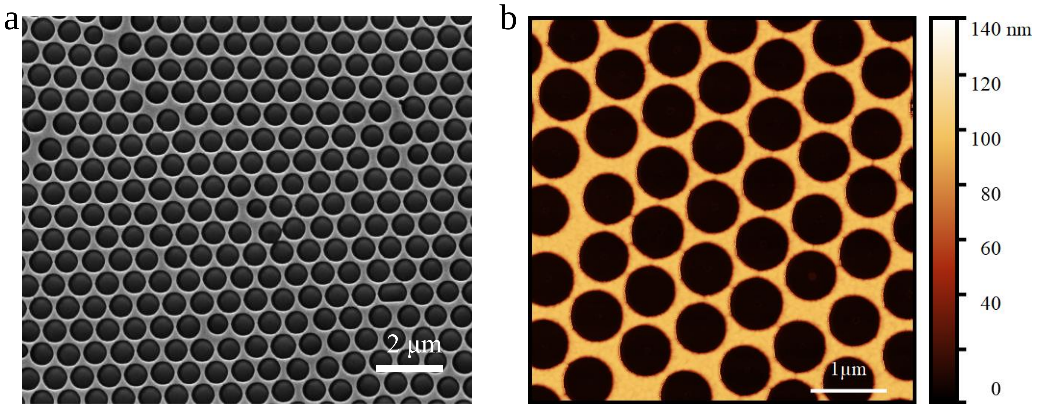

Supplementary Figure 9. a, b, SEM and AFM images of the bare nanomesh electrodes on Al modified SiO_2/Si substrate prepared by polystyrene nanospheres monolayer with the sphere diameter of 800 nm.

7. Vertical devices prepared by supramolecular nanowires

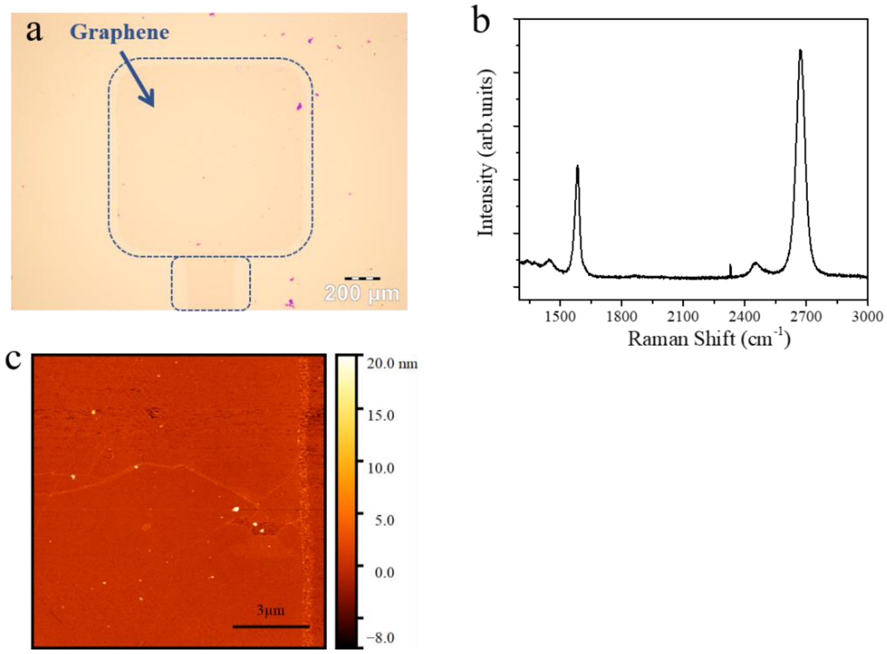

Supplementary Figure 10. a, Optical image of pattern graphene as bottom source electrode. b, Raman spectrum of the monolayer graphene displaying the typical *G* peak at 1585 cm^{-1} and the *2D* peak at 2673 cm^{-1}, with I_{2D}/I_G=2.0. c, Topography AFM image of patterned monolayer graphene.

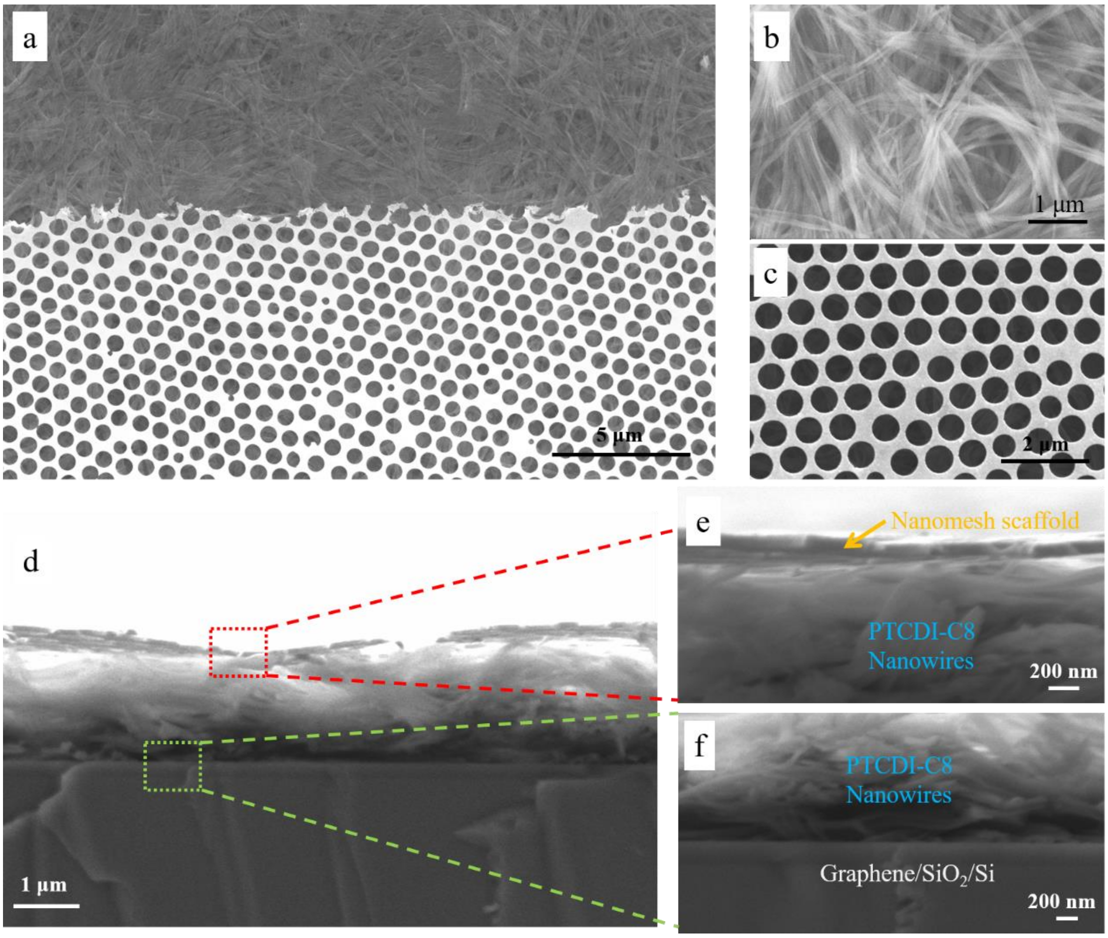

Supplementary Figure 11. a, Large area SEM image of prepared PTCDI-C8 nanowire vertical device with nanomesh scaffold on top of the closely stacked nanowires network. b, c, Zoom-in SEM image of PTCDI-C8 nanowires network and gold nanomesh scaffold on top of PTCDI-C8 nanowires. d, Cross-sectional SEM image of a PTCDI-C8 nanowires based vertical device. e, Zoom-in SEM image of the Au nanomesh scaffold and PTCDI-C8 nanowires network interface. f, Zoom-in SEM image of the PTCDI-C8 nanowires network and Graphene/SiO_2/Si interface.

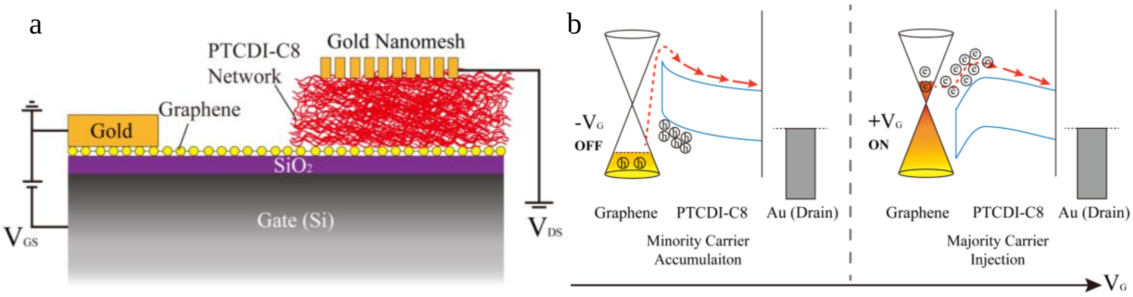

Supplementary Figure 12. a, A schematic of the cross-sectional view of the device with the bottom graphene and top gold nanomesh scaffold as the source and drain electrodes, respectively. The vertically stacked PTCDI-C8 nanowires network working

as the semiconducting channel with its thickness deciding the channel length. b, Proposed band diagrams as a function of V_G associated with a n-type vertical field-effect transistor.

Supplementary Figure 12 b illustrates the band diagrams associated with the PTCDI-C8 VFET. First, due to the partially screened field effect, for $V_G < Vth$, the minority carriers (holes for a n-type VFET) are accumulated in both graphene and at the semiconductor/graphene interface, generating a depletion region that hinders the transport of the majority carriers (electrons). For $V_{th} < V_G < V_{CNP}$ (CNP: charge neutrality point of graphene), the majority carriers are accumulated at the semiconductor/graphene interface, therefore building an inversion layer. However, graphene is still hole rich, thereby limiting the electron injection. For $V_G > V_{CNP}$, the majority carriers are induced both in graphene and at the interface, so a continuous conducting channel is created at the heterointerface[1].

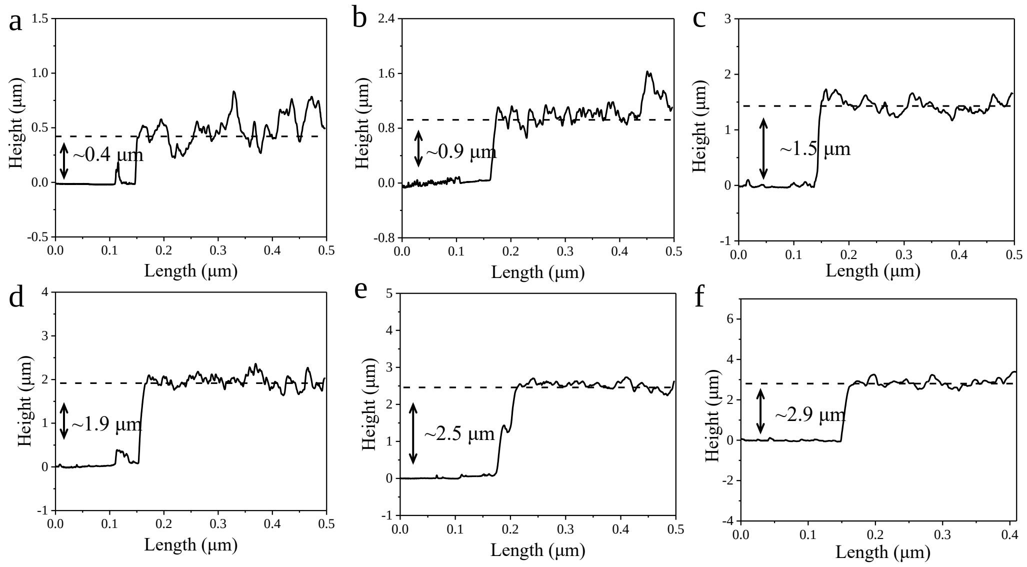

Supplementary Figure 13. Height profiles of PTCDI-C8 nanowires networks measured using a Alpha-Step IQ profilometer. PTCDI-C8 nanowires networks are fabricated through the vacuum-assisted stamp method and controlled by changing volume of PTCDI-C8 nanowire suspension. Average thickness: a, 0.4 μm; b, 0.9 μm; c, 1.5 μm; d, 1.9 μm; e, 2.5 μm; f, 2.9 μm.

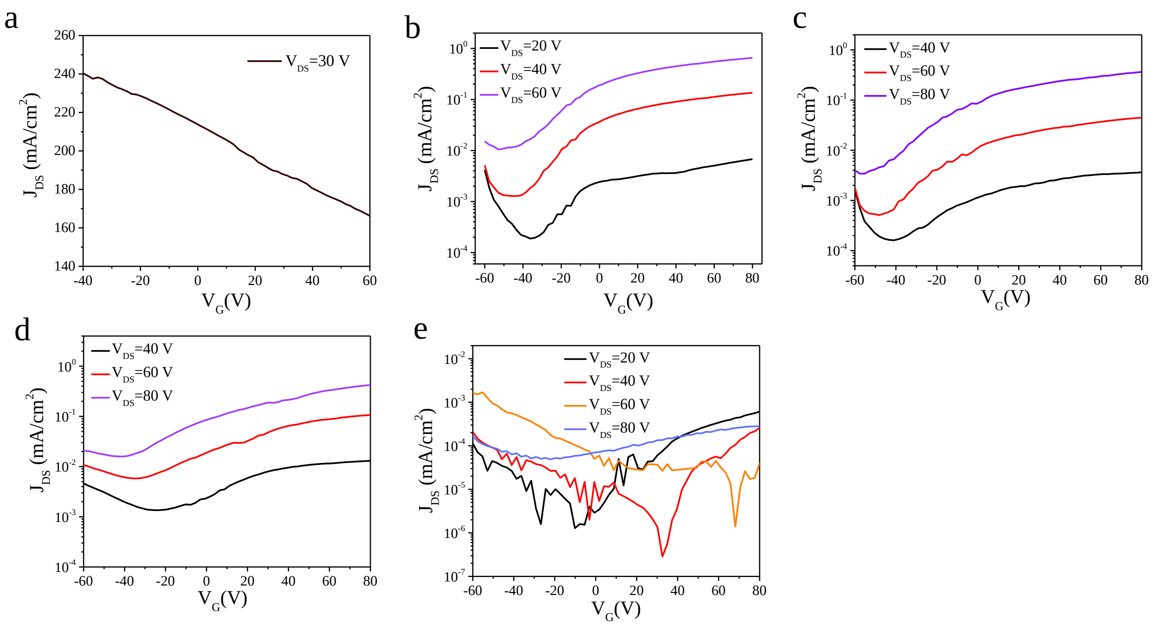

Supplementary Figure 14. Transfer characteristics of the PTCDI-C8 nanowires network VFETs with different channel lengths, after thermal annealing at 120 °C for 30 min. a, 0.4 µm; b, 0.9 µm; c, 1.5 µm; d, 1.9 µm; e, 2.9 µm.

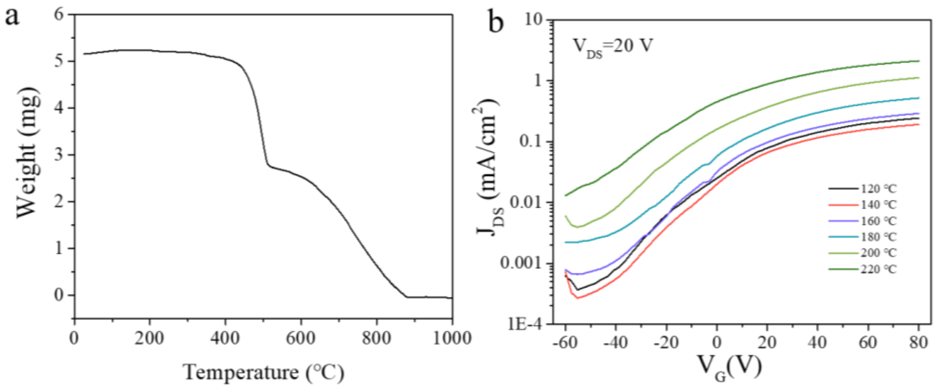

Supplementary Figure 15. a, Thermogravimetric analysis (TGA) of PTCDI-C8. b, Transfer characteristics of the PTCDI-C8 nanowires VFETs at different thermal annealing temperature for 30 min.

The thermal property of PTCDI-C8 was evaluated by thermogravimetric analysis (TGA); the decomposition curves are recorded in the range 25-1000 °C operating under nitrogen atmosphere, with a thermal step of 10 °C/min on a Mettler Toledo TGA/SDTA851e system. From TGA thermogram shown in Supplementary Figure 15 a, PTCDI-C8 shows very good thermal stability with the decomposition temperatures (T_d, 5% weight loss) of 431 °C. The high thermal stability at elevated temperatures of PTCDI-C8 provides an adequate guarantee for practical applications in organic electronics.

Temperature (°C)	J_{ON} (mA/cm^2)	J_{ON}/J_{OFF}
120	0.054±0.023	1.05E+02
140	0.089±0.022	3.28E+02
160	0.138±0.051	2.99E+02
180	0.275±0.088	3.54E+02
200	0.999	8.40E+01
220	2.356	2.85E+01

Supplementary Table 2. Dependence of the on current density (J_{on}: current density at 80V) and current density on/off ratio (J_{on}/J_{off}) on the different annealing temperatures.

VFETs based on full-coverage PTCDI-C8 nanowires network after thermal treatment at 120°C, 140°C, 160°C, 180°C, 200°C and 220°C. Thermal treatment at 220 °C leads to higher current density, yet the on/off ratio degrades from 10^2-10^3 to 29.

8. Vertical phototransistors of supramolecular network based on nanomesh scaffold

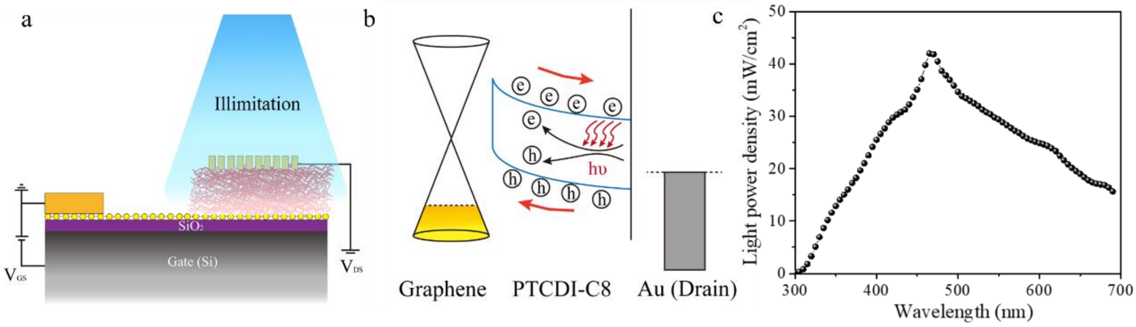

Supplementary Figure 16. a, Schematic illustration as side view of the vertical phototransistor based on PTCDI-C8 nanowires network with top light illumination. b, Band diagram of the vertical phototransistors under illumination. c, Incident light power of monochromatic source (300 nm - 690 nm at 100% intensity) used for the result shown in Figure 3b.

Supplementary Figure 16 a shows the schematic illustration of PTCDI-C8 nanowires network based VPT, with light irradiation coming from the top, being introduced directly onto the active layer of the device through the semitransparent nanomesh scaffold. When the PTCDI-C8 nanowires network was stacked on the graphene, the holes in the latter move to PTCDI-C8 nanowires, resulting in the upward bending of the energy levels near the graphene (Supplementary Figure 16 b), and the formation of the built-in electric field between the graphene/PTCDI-C8 contact (depletion region). When the device is exposed to light, the PTCDI-C8 nanowires absorb the photon forming photoexcited electron-hole pairs. The latter are separated by the source-drain

voltage and the electrons move toward the drain electrode, forming the photocurrent in the circuit[2].

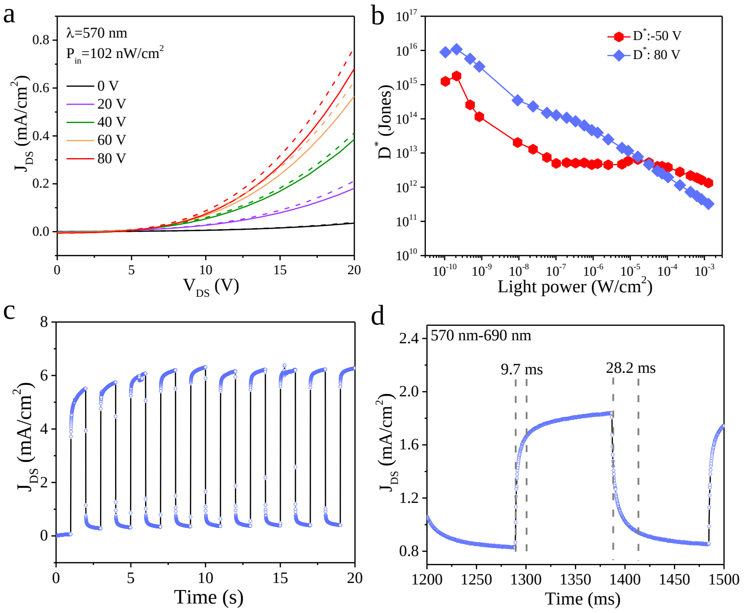

Supplementary Figure 17. a, Output characteristics of the vertical phototransistors with different V_G (black = 0 V, purple = 20 V, green = 40 V, orange = 60 V, and red = 80 V) under dark (solid line) and 102 nW/cm^2 570 nm green light illumination (dashed line). b, Specific detectivity (D^*) versus different irradiances in the depleted area (V_G = -50 V, red diamond line) and the accumulated area (V_G = 80 V, blue hexagon line), the detectivity maximum can reach 6 × 10^{16} Jones attributed to high sensitivity of the PTCDI-C8 nanowires VPTs at 570 nm. c, Optoelectrical switching behavior of the PTCDI-C8 nanowires VPTs under V_{DS} = 20 V, V_G = -50 V, giving a large on/off ratio under an intensity of 33.0 mW/cm^2 at 570 nm. d, Shifting incident wavelength between 570 nm and 690 nm under V_{DS} = 20 V, V_G = -50 V.

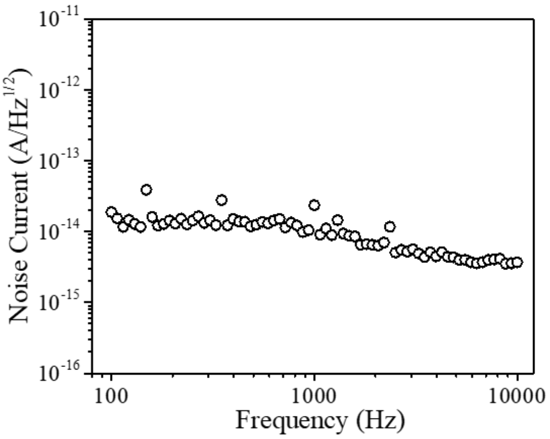

Supplementary Figure 18. Noise current spectra of PTCDI-C8 nanowires VPTs at V_{DS} = 5V.

The total noise current consists of three parts; the shot noise ($i_{n,s}$), thermal noise ($i_{n,t}$) and 1/f noise. 1/f noise is related to lattice scattering, phonon scattering and impurity scattering of materials. Therefore, to reduce the influence of 1/f noise during noise measurement, the bias voltage was selected as 5 V. Shot noise and thermal noise are generally related to quantum effects and thermal effects, respectively. If measured at high frequency (above 1000Hz), usually shot noise is dominant in our devices, thermal noise can be ignored. However, at low frequencies (< 100 Hz), 1/ f noise is usually larger.

These noise types can be expressed as[3]:

$$i_{(n,s)} = \sqrt{2qI_{Dark}B}$$

$$i_{(n,t)} = \sqrt{\frac{4k_B TB}{R}}$$

where q is the elementary charge, I_{Dark} is the dark current, and B is the instrument bandwidth, and the latter has a value related to the bandwidth of the lock-in amplifier, which is 0.833 Hz. K_B is the Boltzmann constant, T is the temperature, and R is the resistance. All measurements were performed at room temperature and the resistance can be obtained from the I-V curves. When the shot noise and thermal noise are calculated separately, the numerical value of the shot noise (63.4 fA/Hz$^{1/2}$) is almost ten times larger than that of the thermal noise (6.7 fA/Hz$^{1/2}$), which demonstrated that the thermal noise is negligible at this time and the total noise is govern by the shot noise.

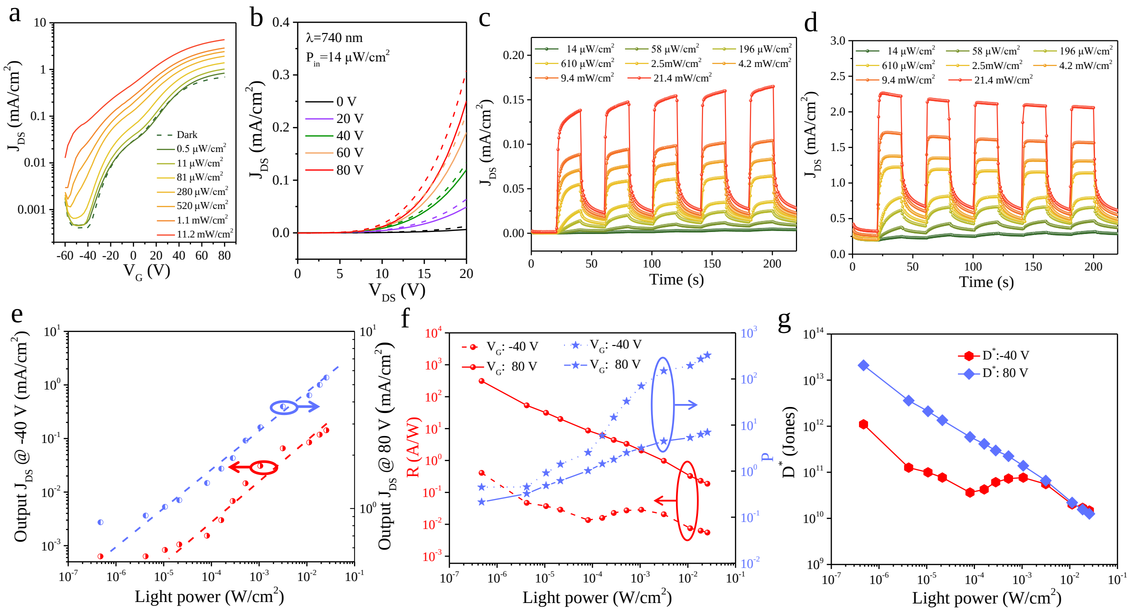

Supplementary Figure 19. a, Transfer characteristics of vertical phototransistors based on nanowires network under 740 nm various irradiances compared to that in darkness, V_{DS} = 20 V. b, Output characteristics of the vertical phototransistors with different V_G (black = 0 V, purple = 20 V, green = 40 V, orange = 60 V, and red = 80 V) under dark

(solid line) and 14 µW/cm^2 740 nm NIR light illumination (dashed line). c, d, Optoelectrical switching behavior of the PTCDI-C8 nanowires VPTs at depleted region (c, V_{DS} = 20 V, V_G = -50 V) and accumulated region (d, V_{DS} = 20 V, V_G = 60 V) under various intensity of 740 nm. e, Photocurrent density value at V_G = -40 V (red dots) and V_G = 80 V (blue dots) plotted against irradiances at 740 nm, the calculated LDR are 61 dB and 76 dB, respectively. f, R and P versus different irradiances in the depleted area (V_{DS} = 20 V, V_G = -40 V, dash line) and in the accumulated area (V_{DS} = 20 V, V_G = 80 V, solid line), the maximum R and P values can reach 3.1 × 10^2 A/W and 3.3 × 10^2 at 740 nm. g, D* versus different irradiances in the depleted area (V_{DS} = 20 V, V_G = -40 V, red diamond line) and the accumulated area (V_{DS} = 20 V, V_G = 80 V, blue hexagon line), the detectivity can reach 2 × 10^{13} Jones at 740 nm.

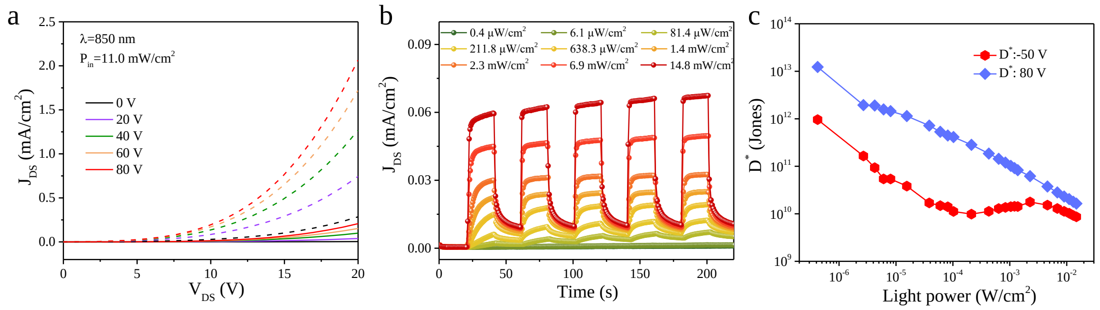

Supplementary Figure 20. a, Output characteristics of the vertical phototransistors based on nanowires network with different V_G (black = 0 V, purple = 20 V, green = 40 V, orange = 60 V, and red = 80 V) under dark (solid line) and 11.0 mW/cm^2 850 nm NIR light illumination (dashed line). b, Optoelectrical switching behavior of the PTCDI-C8 nanowires VPTs at depleted region (V_{DS} = 20 V, V_G = -50 V) under various intensity of 850 nm. c, D* versus different irradiances in the depleted region (V_{DS} = 20 V, V_G = -50 V, red diamond line) and the accumulated region (V_{DS} = 20 V, V_G = 80 V, blue hexagon line), the detectivity can reach 1 × 10^{13} Jones at 850 nm.

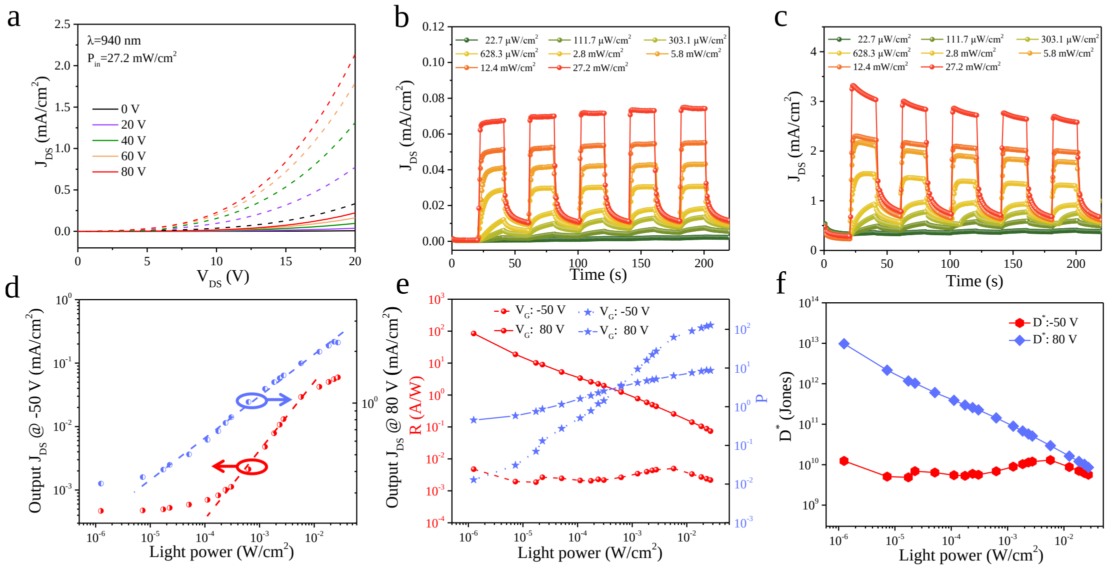

Supplementary Figure 21. a, Output characteristics of the vertical phototransistors based on nanowires network with different V_G (black = 0 V, purple = 20 V, green = 40 V, orange = 60 V, and red = 80 V) under dark (solid line) and 27.2 mW/cm^2 940 nm NIR light illumination (dashed line). b, c, Optoelectrical switching behavior of the PTCDI-C8 nanowires VPTs at depleted region (b, V_{DS} = 20 V, V_G = -50 V) and accumulated region (c, V_{DS} = 20 V, V_G = 60 V) under various intensity of 940 nm. d, Photocurrent density value at V_G = -50 V (red dots) and V_G = 80 V (blue dots) plotted against different irradiances at 940 nm, the calculated LDR are 28 dB and 61 dB, respectively. e, R and P versus different irradiances in the depleted area (V_{DS} = 20 V, V_G = -50 V, dash line) and in the accumulated area (V_{DS} = 20 V, V_G = 80 V, solid line), the maximum R and P values can reach 85 A/W and 1.2 × 10^2 at 940 nm. f, D* versus different irradiances in the depleted area (V_{DS} = 20 V, V_G = -50 V, red diamond line) and the accumulated area (V_{DS} = 20 V, V_G = 80 V, blue hexagon line), the detectivity can reach 1 × 10^{13} Jones at 940 nm.

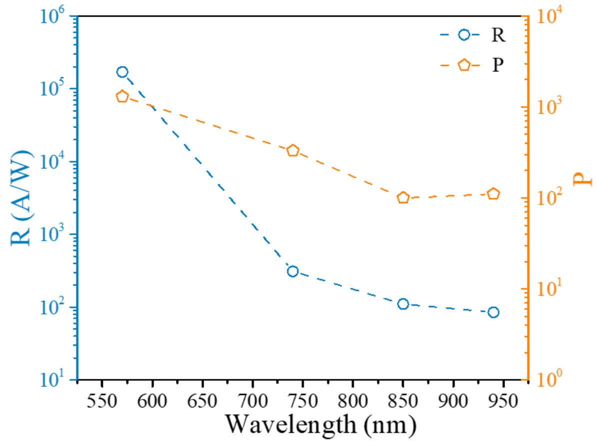

Supplementary Figure 22. Dependence of R and P values on the wavelength.

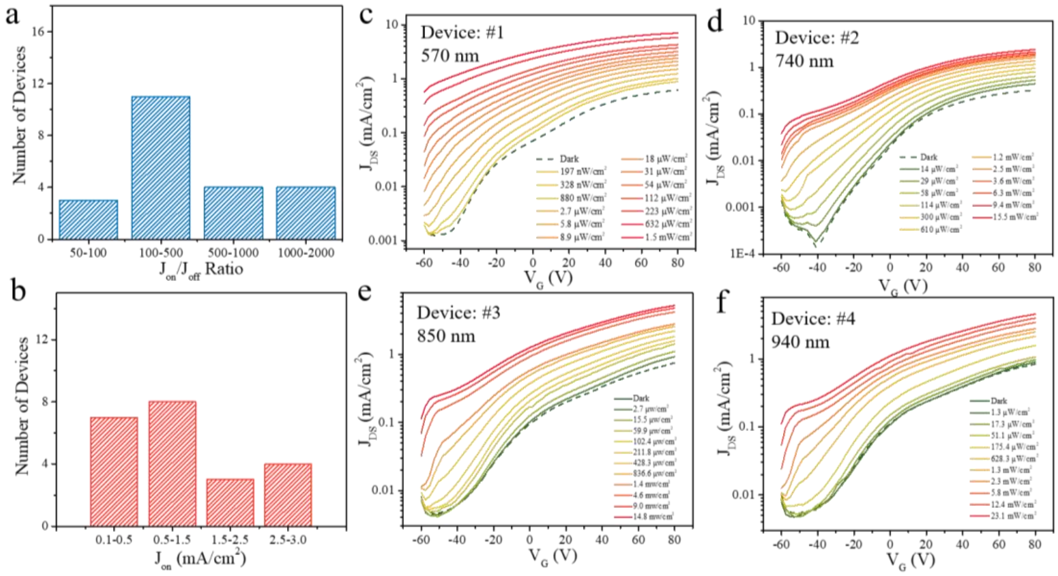

Supplementary Figure 23. a, Device J_{on}/J_{off} ratio, and b, current density distribution based on 22 different PTCDI-C8 nanowires network VPTs. c-f, Transfer characteristics of different batches of vertical phototransistors based on nanowires network under various irradiances.

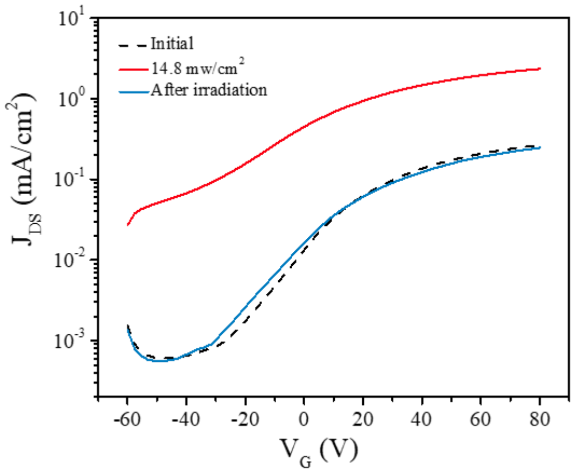

Supplementary Figure 24. Transfer curves of PTCDI-C8 nanowires VPTs with V_{DS} = 20 V before and after 850 nm light irradiation. The device returns to the initial state when the light turned off.

9. Vertical phototransistors of supramolecular network based on continuous gold film

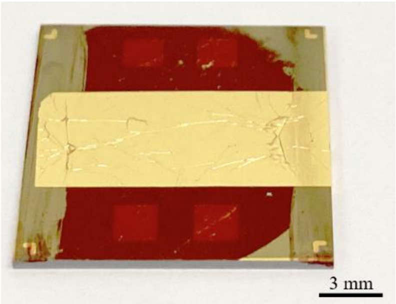

Supplementary Figure 25. Photograph of the PTCDI-C8 nanowires vertical device based on a continuous gold film without any nanostructures.

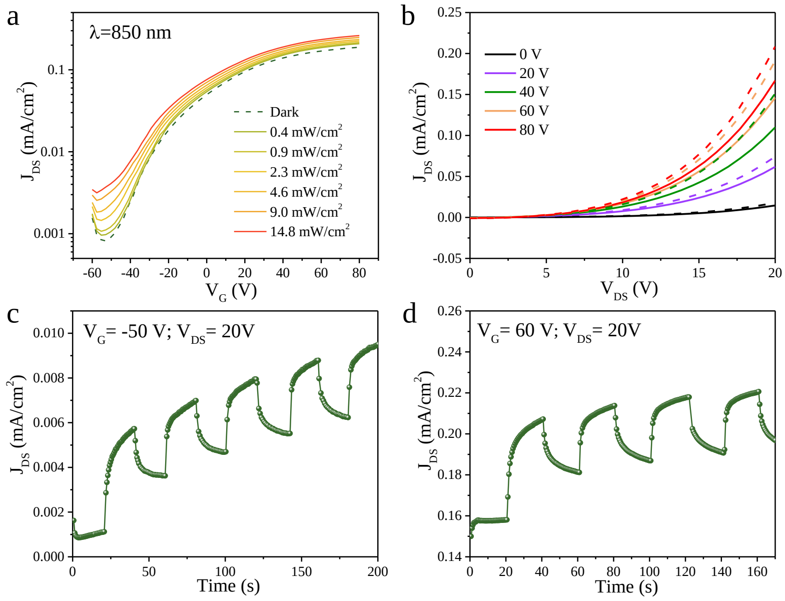

Supplementary Figure 26. a, Transfer characteristics of vertical phototransistors based nanowires network with the continuous gold films as the top electrode (as shown in Supplementary Figure 25) under 850 nm various irradiance compared to that in darkness, V_{DS} = 20 V. b, Output characteristics of the vertical phototransistors with different V_G (black = 0 V, purple = 20 V, green = 40 V, orange = 60 V, and red = 80 V) under dark (solid line) and 10.0 mW/cm^2 850 nm NIR light illumination (dashed line). c, d, Optoelectrical switching behavior of the PTCDI-C8 nanowires VPTs at depleted region (c) and accumulated region (d) under 10.0 mW/cm^2 850 nm NIR light illumination.

10. Supplementary References

1. Shih, C. J. et al. Partially-Screened Field Effect and Selective Carrier Injection at Organic Semiconductor/Graphene Heterointerface. *Nano Lett.* **15**, 7587-7595 (2015).
2. Liu, J. et al. Organic-Single-Crystal Vertical Field-Effect Transistors and Phototransistors. *Adv. Mater.* **30**, 1803655 (2018).
3. Bao, C. X. et al. Low-Noise and Large-Linear-Dynamic-Range Photodetectors Based on Hybrid-Perovskite Thin-Single-Crystals. *Adv. Mater.* **29**, 1703209 (2017).
